# A unifying framework for understanding how edge effects reshape the structure, composition and function of forests

**DOI:** 10.1111/nph.70457

**Published:** 2025-08-13

**Authors:** Rebecca Banbury Morgan, Tommaso Jucker

**Affiliations:** ^1^ School of Biological Sciences University of Bristol 24 Tyndall Avenue Bristol BS8 1TQ UK

**Keywords:** aboveground biomass, edge effects, forest fragmentation, forest structure, patch contrast, remote sensing

## Abstract

Due to widespread deforestation and habitat fragmentation, today most of the world's forests lie within 1 km of an edge. Forests near edges are warmer, drier, receive more light, and are more exposed to wind and other disturbances than intact forests, profoundly altering the demographic processes that drive their dynamics. For reasons that remain poorly understood, the magnitude and direction of edge effects on forest structure, composition and function vary considerably across forest ecosystems. Here, we develop a unifying framework that aims to reconcile these apparently contrasting outcomes to forest edge creation by explicitly accounting for the effects of edge age, climatic context and forest structure. We begin by identifying four stages of forest edge development, arguing that demographic trajectories after edge creation are actually broadly similar across different forest types. We then consider how regional differences in climate and forest structure help explain why in tropical rainforests aboveground biomass typically declines sharply at forest edges, while in cooler climates the opposite is often true. Finally, we outline a series of concrete predictions made by our framework and discuss how these could be tested using ever‐growing archives of remote sensing products that capture ecosystem change across broad spatio‐temporal scales. In doing so, we aim to offer a fresh perspective on a research field that has captivated ecologists for half a century.

**Table:** 

	Contents	
	Summary	529
I.	Introduction	530
II.	Forest edge responses through time	530
III.	Variation in edge effects across environmental gradients	532
IV.	Reconciling variation in forest edge effects on aboveground biomass	533
V.	Forest edges in a human‐modified world	535
VI.	Using remote sensing to characterise forest edge effects across spatial, temporal and ecological scales	536
VII.	Conclusions	538
	Acknowledgements	538
	References	538

## I. Introduction

As forests are cleared for agriculture, timber and development, remaining forests become increasingly fragmented and isolated, and the proportion of forests growing in close proximity to an edge with open habitat increases (Fischer *et al*., 2021; Ma *et al*., 2023). Globally, anthropogenic land clearance is estimated to have left *c*. 70% of the world's forests within 1 km of an edge and *c*. 20% within just 100 m of one of these transition zones (Haddad *et al*., 2015). Forest edges are exposed to distinct environmental conditions compared to forest interiors, including higher temperatures, wind speeds and light availability, and lower water availability (Ewers & Banks‐Leite, 2013; Magnago *et al*., 2015), all of which significantly impact forest function.

Ecologists have been studying habitat fragmentation and its impacts on forest ecosystems for decades. Across a landscape, ecosystem fragmentation creates a patchwork of intact habitats embedded within a heavily transformed matrix. These are connected by a network of edges where intact forests and the surrounding matrix meet, forming abrupt ecological transition zones with altered ecosystem structure and functioning (Cadenasso *et al*., 2003; Schmidt *et al*., 2017). Previous frameworks have attempted to both conceptualize how physical processes vary across edges and to develop ways of quantifying these changes, with particular focus on forest edges (Harper *et al*., 2005; Ewers & Didham, 2006). This work has provided a strong foundation for empirical research into the biophysical and ecological outcomes of forest edge creation across a wide variety of ecosystems (Fischer *et al*., 2021; Morreale *et al*., 2021; Ordway & Asner, 2020; Pfeifer *et al*., 2017; Silva *et al*., 2020; Willmer *et al*., 2022). However, we currently lack a conceptual framework that reconciles the growing body of research showing that edge effects can vary considerably in their strength across different forest ecosystems.

Following the creation of an edge, clear shifts in forest structure, composition and function typically occur. However, the magnitude, and even direction, of these shifts can vary substantially among forest types. In tropical forests, edge effects are associated with marked declines in aboveground biomass (AGB) that can extend several hundreds of meters into the forest interior (Ordway & Asner, 2020; Anderson *et al*., 2022; Bauer *et al*., 2024). By contrast, in temperate forests AGB has been found to remain unchanged (Ziter *et al*., 2014) or even increase at forest edges (Reinmann & Hutyra, 2017; Meeussen *et al*., 2021; Morreale *et al*., 2021). Despite these seemingly opposing outcomes to forest edge creation, many of the underlying processes that ultimately govern shifts in AGB are remarkably consistent across different forest types (Table 1). For example, edges in both temperate and tropical regions display increases in stem densities (Ziter *et al*., 2014; Morreale *et al*., 2021; Maeda *et al*., 2022), declines in canopy height (Almeida *et al*., 2019; Meeussen *et al*., 2020), declines in the frequency of late‐successional, shade‐tolerant species (Ziter *et al*., 2014; Dantas de Paula *et al*., 2015; Qie *et al*., 2017) and increases in both productivity and stem turnover (Reinmann & Hutyra, 2017; Laurance *et al*., 2018; Morreale *et al*., 2021; Bauer *et al*., 2024). This suggests that while the outcomes for AGB may differ across forest types, the underlying responses to edge formation are similar, providing an opportunity to develop a unifying framework for predicting edge effects across forest types.

**Table 1:** Comparisons of forest structural, compositional and functional attributes between forest edges and interiors.

		Forest interior	Forest edge
Forest structure	Canopy height and density	↑	**↓**
	Mean tree size	**↑**	**↓**
	Stem density	**↓**	**↑**
	Frequency of large trees	**↑**	**↓**
Forest composition	Proportion of late‐successional species	**↑**	**↓**
	Proportion of shade‐tolerant species	**↑**	**↓**
	Wood density	**↑**	**↓**
Forest function	Tree growth rates	**↓**	**↑**
	Tree mortality rates	**↓**	**↑**
	Biomass turnover rate	**↓**	**↑**

Arrows indicate relatively higher (**↑**) and lower (↓) values.

In this review, we offer a framework for understanding variation in forest edge responses through time and across environmental gradients. Based on observed consistencies in forest edge effects on stand structure, we first outline a framework describing forest edge development over time following edge creation. We then use the concept of *patch contrast* – which predicts that the strength of edge response is related to the magnitude of the structural and compositional differences between the ecosystems on either side of an edge (Harper *et al*., 2005) – to consider major drivers of variation in edge responses across global gradients in climate and forest structure. Next, we integrate these ideas to illustrate how we can explain variation in edge effects on AGB across ecosystems by accounting for differences in climate, forest structure and edge age. In doing so, we also explore how edge effects might manifest in novel human‐modified ecosystems recovering from disturbance, such as secondary forests regenerating after clearing and edges created through forest expansion as opposed to loss. Finally, we outline a set of predictions made by our framework and suggest approaches for testing them using increasingly available remote sensing datasets acquired across varying spatial, temporal and ecological scales.

In developing this framework, we consider only ecosystems that are dominated by trees, excluding arid ecosystems where trees form a scattered presence across the landscape or savannas where they are co‐dominant with grasses. This encompasses forests and woodlands spanning a broad range of climates and exhibiting a wide range of structural characteristics, including substantial variation in canopy cover, height and complexity. We focus primarily on edges formed and maintained through anthropogenic activities, though we expect that aspects of the framework will also be applicable to persistent natural edges, such as those associated with water bodies or geological formations.

## II. Forest edge responses through time

**Fig. 1:**
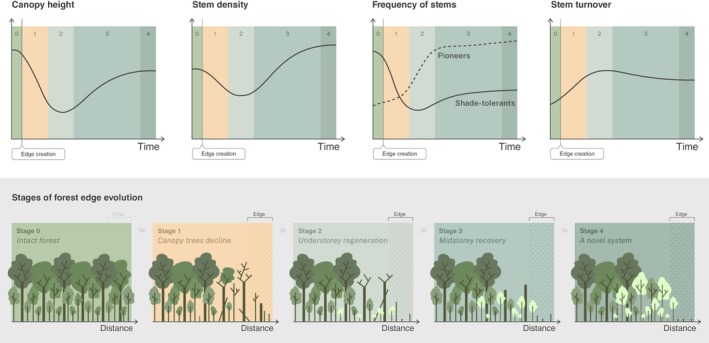
Graphical illustration of the four key stages of edge evolution following edge creation relative to initial starting conditions in an intact tropical rainforest (stage 0). The top panels show predicted changes in canopy height, stem density, functional composition and stem turnover rates across the four stages of edge evolution.

Forest edge responses are not static but change through time (Laurance *et al*., 2018), following a series of dynamic processes that unfold over multiple years and decades (Fig. 1). This makes direct comparisons of edge responses across different sites inherently challenging, as they can easily be confounded by the effects of edge age (Ordway & Asner, 2020). Consequently, understanding edge effects on forest structure, composition and function develop through time is an essential first step to contextualizing the wide range of responses that have been reported in the literature. Here, we synthesize evidence from studies of forest edge responses across tropical, temperate and boreal regions to outline a framework of forest edge development through time.

In comparison with nonforested ecosystems and adjacent areas of cleared land, mature intact forests have a more complex three‐dimensional structure, with higher vertical complexity, canopy cover and AGB. The majority of AGB is concentrated in the largest stems (Lutz *et al*., 2018), which tend to dominate the canopy, and typically have relatively low mortality rates (Esquivel‐Muelbert *et al*., 2020). Forest canopies create a buffer between the external environment and the forest interior, and as a result, forests have internal microclimates that are distinct from those outside the canopy, with lower temperatures and higher humidity in the forest interior than in the exterior (Ewers & Banks‐Leite, 2013; De Frenne *et al*., 2021; Meeussen *et al*., 2021).

When forest edges form, the abiotic conditions that trees are exposed to are significantly altered. Forest edges have higher light availability and are exposed to higher wind speeds, particularly where transitions from the matrix to the forest edge are sharp (Magnago *et al*., 2015; Smith *et al*., 2018). Higher outside air temperatures lead to increased evapotranspiration, resulting in decreases in soil water availability and increases in vapour pressure deficit (VPD), which increase water stress for trees at forest edges (Magnago *et al*., 2015; Nunes *et al*., 2022). Nutrient deposition and cycling are also altered near edges, with declines in soil carbon content, but increases in inorganic nitrogen, calcium and magnesium (Weathers *et al*., 2001; Garvey *et al*., 2023).

In response to these environmental stressors, mortality rates typically increase following edge creation (Harper *et al*., 2005; Smith *et al*., 2018). Large canopy trees are the most affected, as they are most vulnerable to wind throw and drought (Bennett *et al*., 2015; Bell *et al*., 2017; Lindenmayer & Laurance, 2017; Stovall *et al*., 2019). The immediate impact of this is seen in declines in many key stand structural parameters, including canopy height and stem density, and increases in canopy gap frequencies (Nascimento & Laurance, 2004; de Paula *et al*., 2011; Yoshida *et al*., 2011; Laurance *et al*., 2018).

These initial canopy losses exacerbate the change in abiotic conditions near the edge, further increasing light availability to the understorey, which drives rapid growth of saplings in gaps. Species with high growth rates and low wood densities, which are able to respond rapidly to high resource availability, recruit best in these conditions. The result is a shift in species composition away from species associated with old‐growth forests and towards communities dominated by pioneer and habitat generalist species (Laurance *et al*., 2006a,b; Pütz *et al*., 2011; Ziter *et al*., 2014; Melito *et al*., 2018). In addition, particularly within tropical regions, liana abundance increases in disturbed forests near edges (Ngute *et al*., 2024), leading to higher rates of tree mortality and suppressing tree growth (Laurance *et al*., 2014; van der Heijden *et al*., 2015; Campbell *et al*., 2018).

At this point, increases in growth and recruitment of stems at forest edges start to drive some recovery of stand structure, with recruitment within gaps leading to increased canopy cover, canopy height and stem densities (de Paula *et al*., 2011; Tomimatsu *et al*., 2015; Laurance *et al*., 2018; Almeida *et al*., 2019). However, the forest is now compositionally, structurally and functionally distinct from the original interior forest. Stands at forest edges tend to shift away from a low turnover system towards one characterized by higher dynamism, with elevated growth, mortality and recruitment rates driven by the faster life histories of early successional species (Santos *et al*., 2008; Tabarelli *et al*., 2008; Laurance *et al*., 2011).

Taken together, this body of work suggests consistency in the trajectories of forest edge development across forest types, with the most pronounced structural changes occurring in the years immediately following edge creation (Silva *et al*., 2020). Initial losses of large, canopy‐dominant trees are expected to saturate over time (Dantas de Paula *et al*., 2015; Ordway & Asner, 2020) and some recovery may then occur (Harper *et al*., 2015; Tomimatsu *et al*., 2015; Almeida *et al*., 2019). However, forest edge communities remain distinct from those of forest interiors, even many decades after edge creation (Tomimatsu *et al*., 2015; Almeida *et al*., 2019; Ordway & Asner, 2020; Anderson *et al*., 2022). Based on this evidence, we outline four key stages in forest edge development (Fig. 1):

1. Canopy tree decline: the initial impact following edge creation, characterized by rapid declines in canopy height, stem density and mean tree size, as well as increases in canopy gaps.
2. Understorey regeneration: the inflexion point of edge response, where the initial structural impacts of edge creation are met with increases in recruitment and sapling growth, resulting in an increase in understorey stem density.
3. Midstorey recovery: rapid growth of early‐successional species leads to some recovery of canopy height, increased frequencies of mid‐sized stems and filling in of canopy gaps.
4. A novel system: the system stabilizes into a novel state, and is now characterized by higher stem turnover rates, higher stem densities and a higher proportion of early successional species characterized by resource‐acquisitive strategies.

## III. Variation in edge effects across environmental gradients

To understand why edge effects are stronger in some places than others, it is important to also consider how much the creation of the edge alters the environmental conditions within which trees grow. This is conceptualized in the idea of patch contrast, which is defined as ‘the difference in composition, structure, function, or microclimate between adjoining ecosystems on both sides of the edge’ (Harper *et al*., 2005). Where patch contrast is higher, abiotic gradients from forest interior to edge are steeper, and so the impact of edge creation is expected to be greater (Harper *et al*., 2005). There are a myriad of factors that can affect patch contrast, and these can operate across multiple spatial and ecological scales. On a local scale, ideas of patch contrast help to explain variation in the magnitude of edge effects within a landscape. For example, forest edges on topographically exposed ridges may be more vulnerable than those in gullies or near rivers (Bell *et al*., 2017; Nunes *et al*., 2021). On regional and global scales, patch contrast helps to explain observed differences in the outcomes of edge responses across forest types (Harper *et al*., 2005), including the higher strength of edge effects in tropical regions compared with temperate regions (e.g. Morreale *et al*., 2021; Bauer *et al*., 2024).

Extending the framework put forward by Harper *et al*. (2005), we suggest that a theory of patch contrast offers a valuable starting point for exploring global gradients in edge responses. Specifically, we identify four interlinked climatic and forest structural attributes which we expect to be foundational in determining large‐scale patterns of patch contrast: (1) temperature, (2) water availability, (3) canopy density and (4) canopy height. This framework will facilitate moving beyond individual studies to consider edge responses as a function of gradients in climate and structure.

The buffering effect of forest canopies strongly modulates the microclimate of intact forests, resulting in marked abiotic gradients when transitioning from the forest's interior to its edge. Two microclimatic gradients are likely to be particularly important in shaping patch contrast at large spatial scales: temperature and water availability. In warm tropical regions, interior forest temperatures are typically several degrees cooler than the outside air temperature (Ewers & Banks‐Leite, 2013; Hardwick *et al*., 2015; De Frenne *et al*., 2019), creating a strong temperature gradient between the forest interior and the outside matrix. However, the buffering effect of forest canopies has been found to decline at lower temperatures, with forest interior temperatures in cooler regions being much more similar to the outside air temperature (De Frenne *et al*., 2019; Meeussen *et al*., 2021). This suggests the potential for a strong latitudinal and elevational gradient in temperature‐related patch contrast across global forests.

The potential influence of water availability on patch contrast is more complex, in part because of interactions between temperature, water availability and demand. Relative humidity in forest interiors is typically higher than at edges, which, combined with lower temperatures, leads to lower VPD (Didham & Ewers, 2014; Magnago *et al*., 2015; Schmidt *et al*., 2017). In addition, temperature buffering in forest interiors declines with decreasing water availability, as evaporative cooling also declines (De Frenne *et al*., 2021). As a result, microclimate contrast between forest interiors and edges is highest where water availability is high and forest interiors have high humidity and low VPD. These contrasts may be further exacerbated by the fact that soil water availability declines at forest edges (Nunes *et al*., 2022), while tree water use increases (Herbst *et al*., 2007; Kunert *et al*., 2015), putting trees under increased atmospheric and soil water stress. Drought‐related declines in forest productivity have been shown to be larger at forest edges compared to forest interiors (Schwartz *et al*., 2019) and based on patch contrast, we would expect these declines to be most pronounced in warm regions, where high temperatures are likely to exacerbate evapotranspiration and VPD at forest edges.

Large‐scale climatic drivers of patch contrast help explain large‐scale differences in edge responses among forest types. In the wet tropics, where patch contrast is high, forest edges have been repeatedly shown to be shorter and hold 20–25% less AGB compared to forest interiors (Chaplin‐Kramer *et al*., 2015; Ordway & Asner, 2020). By contrast, much weaker edge effects have generally been observed in cooler temperate and boreal regions (Franklin *et al*., 2021; Harper *et al*., 2005, 2015; Smith *et al*., 2018). The strength of edge effects in temperate regions appears to be further modulated by water availability. Previous work has shown that edge effects tend to be weaker in drier forests relative to moist ones (Harper *et al*., 2005; Chaplin‐Kramer *et al*., 2015). Similarly, declines in canopy height and increases in stem density at forest edges are largest in areas with higher humidity across European forests (Meeussen *et al*., 2020).

Forest structure also varies along climate gradients and is likely to be an important – and confounding – factor in understanding spatial variation in edge responses, as it too is key in determining patch contrast (Harper *et al*., 2005). Dense canopies buffer interior temperatures more strongly than open canopies (Jucker *et al*., 2018c; Zellweger *et al*., 2019), and as a result, the interior microclimates of open‐canopy forests are more similar to conditions outside the forest than the microclimates of closed‐canopy forests (Meeussen *et al*., 2021; John *et al*., 2024). This suggests that differences in forest structure will directly contribute to higher patch contrast in tall, closed‐canopy forests, and as such, we expect that the magnitude of edge effects will broadly increase with canopy density and height. It is worth noting that in forests that are partly or completely deciduous – typical of temperate regions and the dry tropics – the buffering effect of the forest canopy, and by extension patch contrast, will vary seasonally (Zellweger *et al*., 2019). However, we expect that the effect of patch contrast on edge responses will be strongest during the growing season, when trees are responding most directly to their environment, and so any direct effect of deciduousness on edge responses is likely to be small.

Other factors may also explain the direct influence of forest structure in shaping edge responses. For example, tall, closed‐canopy forests have a greater proportion of large trees, which are more vulnerable to wind throw and generate bigger canopy gaps when they fall (Reis *et al*., 2022; Jackson *et al*., 2024). Further, closed‐canopy forests support a high proportion of shade‐tolerant species (Ehbrecht *et al*., 2021), and so shifts in species composition towards a higher proportion of early‐succession species are expected to be more pronounced than in forests with more open canopies.

Climate and forest structure are, of course, interlinked, and most temperate, boreal and tropical dry forest ecosystems have higher canopy openness and a lower stature than tropical moist forest. As a result, the effects of climate and forest structure may compound each other in determining the interior‐to‐edge contrast. Disentangling the relative contribution of these two drivers will always prove a challenge and will require identifying regions where forest structure varies independently of climate. For example, local gradients in soils and topography can drive significant variation in canopy structure within the same climatic region (Jucker *et al*., 2018b). Comparing across regions, dipterocarp forests in Southeast Asia and the forests of the Guiana Shield have higher‐than‐expected canopy heights for their climate (Feldpausch *et al*., 2012), which could make them uniquely vulnerable to edge effects.

Finally, there is an additional interaction to consider between forest structure and light limitation. Increased light availability at forest edges is important in explaining the increased growth rates observed as forest edges develop, and the shift in species composition towards light‐demanding pioneers (Dantas de Paula *et al*., 2015; Reinmann & Hutyra, 2017; Bauer *et al*., 2024; Fig. 1). Light limitation is highest in dense, closed‐canopy forests and at high latitudes, and so we might expect the strength of growth releases at forest edges to be greatest in these environments. This may increase the magnitude of growth‐related edge responses, such as increases in stem density or recovery of canopy height, and is likely to be an important component in explaining observed patterns of increases in stem density and AGB in many temperate forests (Reinmann & Hutyra, 2017; Meeussen *et al*., 2020; Morreale *et al*., 2021). There is no clear evidence of increases in stem density or biomass at boreal forest edges (Jönsson *et al*., 2007; Harper *et al*., 2015). However, these forests typically have shorter, more open canopies and, despite being located at higher latitudes, may be less light‐limited than forests with denser canopies at lower latitudes (Harper *et al*., 2015). This further highlights the importance of interactions between forest structure and environmental factors in shaping edge responses.

## IV. Reconciling variation in forest edge effects on aboveground biomass

**Fig. 2:**
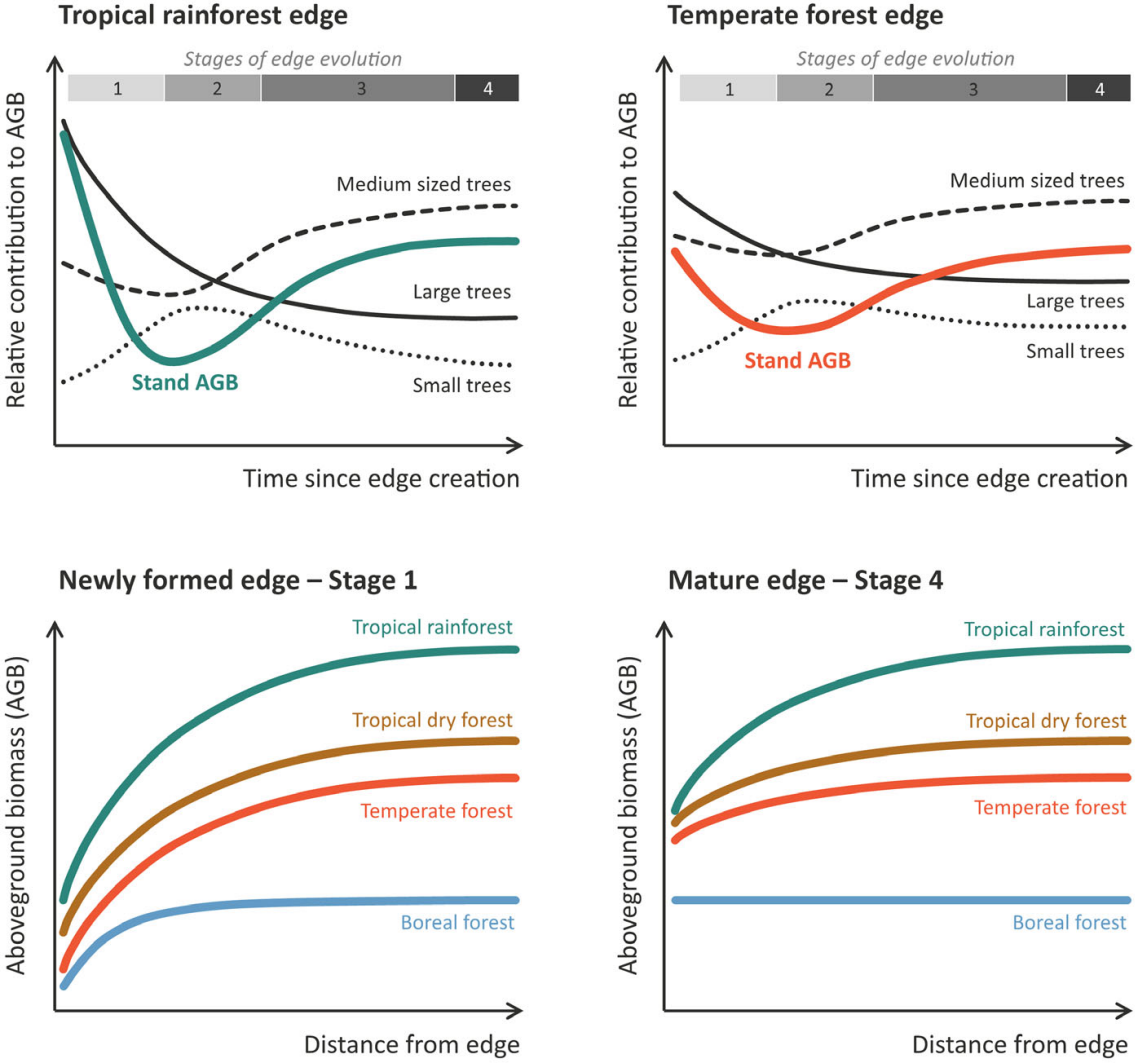
Hypothesized changes in aboveground biomass (AGB) following edge creation across major forest types. The top panels show how the relative contribution of large, medium and small‐sized trees to stand‐level AGB shifts following edge creation in tropical and temperate forests, giving rise to differing temporal AGB trajectories at forest edges in these biomes. The bottom panels show predicted changes in AGB with distance from the edge across a range of climatically contrasting forest types for both recently created edges and ones that have had time to reach a new equilibrium.

We have considered spatial and temporal variation in edge responses through the lenses of patch contrast and the four stages of forest edge development. We now bring these ideas together to explore how patch contrast – as determined by climatic context and baseline forest structure – mediates the development trajectories of forest edges through time. We focus specifically on how edge effects impact stand‐level AGB as an integrative measure of forest structure, and in doing so, attempt to explain why changes in AGB at forest edges can vary so considerably among forest types depending on their climate and size structure (Fig. 2).

The ABG of individual trees increases approximately exponentially with stem diameter (Chave *et al*., 2005). As a result, in intact forests, the majority of the AGB is stored in the largest stems (Fauset *et al*., 2015; Lindenmayer & Laurance, 2017; Lutz *et al*., 2018), even though they make up only a tiny fraction of the trees in the stand. However, following edge creation, there are changes both in the overall stand AGB and in how AGB stocks are distributed among different tree size classes. We can understand these AGB changes through the framework of stages of edge development (Fig. 2). In stage 1, the AGB contribution of the largest stems declines abruptly due to their increased mortality. Because these large trees contribute disproportionately to total AGB, even small declines in their numbers can result in significant AGB losses. In stage 2, regeneration in gaps begins, further increasing the fraction of AGB held in small understorey trees. In stage 3, continued growth of the new cohort of regenerating trees and midstorey recovery begin to shift most of the AGB towards mid‐sized trees. Concurrently, mortality rates of surviving large trees stabilize. Finally, in stage 4, the distribution of AGB across size classes reaches a new equilibrium. The contribution of large stems to overall stand AGB is lower relative to intact forest, and the largest contribution to stand AGB now comes from medium‐sized trees.

Despite the commonality of the underlying processes, the net outcome for stand AGB can vary substantially among forest types (Chaplin‐Kramer *et al*., 2015; Morreale *et al*., 2021). To understand why this is, we need to consider variation in how AGB is distributed across size classes in different forest types. In moist tropical forests, we typically observe a broad spread of stem diameters, with a long tail of large stems. AGB dynamics are dominated by a relatively small number of very large trees, with the largest 1% supporting up to 75% of the total stand AGB (Lutz *et al*., 2018; Piponiot *et al*., 2022). Patch contrast in the moist tropics is generally high, and so edge formation is expected to significantly increase mortality rates of large canopy trees. Because these trees are dominant in shaping the structure of the forest (Farrior *et al*., 2016; Lutz *et al*., 2018; Jackson *et al*., 2024) their losses result in significant declines in AGB and a pronounced reorganization of the distribution of AGB across size classes (Fig. 2a). Notably, we expect to observe a significant decline in the relative AGB contribution of large stems.

By contrast, in temperate and boreal forests, stem diameter distributions typically cover a smaller range of stem sizes, with fewer very large trees. The largest 1% of stems typically hold comparatively less of the total stand AGB (25–40%), with the majority instead stored across a relatively larger number of medium and smaller‐sized stems (Lutz *et al*., 2018; Piponiot *et al*., 2022). Moreover, in temperate forests, patch contrast is lower, and as a result, the mortality effect on canopy stems is less extreme than in tropical forests. Combined together, the lower contribution of large trees to stand AGB and their less pronounced increase in mortality result in a less significant restructuring of AGB across tree size classes. As with tropical forests, we still expect to see increases in the relative AGB contributions of small and medium diameter stems through time, with small stem AGB peaking during the understorey regeneration phase (stage 2 in Fig. 1), and medium stem AGB peaking and stabilizing as the system approaches a new stable state (stage 4 in Fig. 1). However, the magnitude of these changes should be lower in temperate and boreal forests (Fig. 2).

Using this framework to consider outcomes for AGB stocks at forest edges, it becomes clear that moist tropical forests face a double‐edged sword from the interaction between their climatic context and baseline forest structure. They store much more AGB to start with (and therefore have more to lose) and much a higher proportion of AGB stocks is held in a small number of very large trees. They are also highly vulnerable to losing AGB to canopy dieback near edges because their tall, dense canopies create a strong patch contrast in the humid tropical climate. The opposite is true for forests at higher latitudes. Here, edge effects are minimized because the combination of lower, more open forest canopies and cooler temperatures creates weaker patch contrasts (Harper *et al*., 2005). As a result, increases in canopy stem mortality are smaller. Moreover, average stem diameters are smaller, and AGB is less concentrated in the largest trees (Lutz *et al*., 2018), so any increases in large tree mortality have an overall smaller impact on stand AGB.

Regrowth in the understorey and midstorey following edge creation (stages 2–3 in Fig. 1) will offset some of the AGB losses associated with the mortality of canopy‐dominant trees. However, the degree to which this growth response compensates for the increases in mortality will again depend on the interaction between climate and forest structure. In moist tropical forests, high patch contrast will continue to limit the ability of regrowing trees to attain the heights of trees in interior forests long after edge creation. Stem densities and AGB contributions of smaller stems will generally increase, but this fails to compensate for the decline in larger stems (Qie *et al*., 2017; Anderson *et al*., 2022; Maeda *et al*., 2022). By comparison, in regions of lower patch contrast, growth responses are more likely to fully compensate for the relatively smaller increases in the mortality rates of large trees (Morreale *et al*., 2021). Further, because AGB is more equally distributed across size classes, increases in the density of smaller trees can be of a similar magnitude or even exceed losses linked to canopy mortality at forest edges (Ziter *et al*., 2014; Reinmann & Hutyra, 2017). This helps explain why we typically observe significant declines in AGB at tropical forest edges (Ordway & Asner, 2020; Anderson *et al*., 2022; Bauer *et al*., 2024), while in temperate and boreal regions, AGB changes are much smaller and are often positive in forest edges that have reached a new equilibrium (Ziter *et al*., 2014; Harper *et al*., 2015; Meeussen *et al*., 2021; Morreale *et al*., 2021).

Our framework highlights the importance of considering edge age when comparing forest edge responses across and within forest types. This is complicated by the fact that variation in forest edge age is not evenly distributed across the globe. While temperate forest regions are the most highly fragmented (Haddad *et al*., 2015), edges in these regions are typically older and the creation of new forest edges is relatively low (Ma *et al*., 2023). By contrast, tropical regions have undergone rapid fragmentation in recent decades, and have a high concentration of recent edges (Fischer *et al*., 2021; Ma *et al*., 2023). As a result, observed variation in forest edge responses across different studies may be, at least in part, a result of variation in edge age. Temperate forest edges are likely to be significantly further along in their recovery trajectory towards a new equilibrium than tropical forest edges, many of which will still be experiencing the immediate impacts following edge creation.

## V. Forest edges in a human‐modified world

Our framework has so far focused on edges formed and maintained by clearance of mature forests. However, in the real world, there are multiple complexities that may modify or alter edge responses and complicate some of the predictions of our framework. In particular, because of humanity's growing footprint on forests, edges are increasingly being formed through pathways that do not conform to this model. Here we explore two particularly notable examples – edges in regenerating secondary forests and those created via forest expansion – although we note there are others that would fall into this category.

### 1. Edge effects in secondary and degraded forests

A growing proportion of the world's forests are recovering from some form of disturbance or degradation (Pan *et al*., 2024). While the potential buffering effect of secondary forest regrowth on old‐growth forest edges has been studied (Dovčiak & Brown, 2014; Mesquita *et al*., 1999; Smith *et al*., 2023), there is a significant gap in the literature with regard to edge effects within secondary and degraded forests themselves (but see da Silva *et al*., 2024). Secondary and selectively logged forests typically have distinct structural characteristics and species compositions in comparison with mature intact forests, becoming more structurally similar to mature forest as they age (Poorter *et al*., 2021a; Fuentes‐Montemayor *et al*., 2022; Rosen *et al*., 2024). As a result, we expect that trajectories of edge development and the magnitude of edge responses may be modified by the effect of forest age, especially as degraded forests are often locked in a cycle of repeated human disturbance that prevents their full recovery (Bousfield & Edwards, 2025).

Differences in the structure of younger forests will influence patch contrast between the interior forest and the matrix, altering the magnitude of edge effects. Secondary forests generally have shorter, more open canopies and a less complex vertical structure than mature forests, meaning that the microclimate gradient and overall patch contrast from interior to exterior are smaller (Baker *et al*., 2014; Jucker *et al*., 2018c). As a result, we may expect smaller edge effects in younger forests, with these developing in strength as forests age. Comparison of edge effects across forests of differing ages and structural complexity within the same landscape could help to disentangle the importance of forest structural characteristics from climate drivers in shaping edge responses.

Secondary forests are also compositionally distinct from mature ones, with a higher proportion of early‐successional species (Both *et al*., 2019; Poorter *et al*., 2021b). These species are more adapted to disturbance and able to respond rapidly to increases in light availability. As a result, growth responses in younger forests during stages 2 and 3 of edge development (Fig. 1) may be more rapid in secondary forests and result in quicker recovery of AGB. Given that temperate regions have a higher proportion of secondary forest than the tropics, any comparison between the strength of edge effects across biomes would need to carefully consider the confounding effects of forest age.

### 2. Edge formation through forest expansion

Our review has centred on edges formed through forest clearance, reflecting the focus of the scientific literature on the impacts of forest fragmentation on intact forests. However, forest edges are increasingly being created through the opposite process of forest expansion, whether through natural regeneration or planted reforestation schemes (Ma *et al*., 2023). The creation of new edges through forest expansion is particularly common in temperate regions, where historic forest cover has been relatively low and regenerating secondary forests are a key part of the landscape.

We expect that the edge development of a regenerating forest is distinct from that of a cleared edge. First, the development of young, secondary forests is strongly influenced by distance from mature forest due to dispersal limitations. Consequently, stands regenerating in the proximity of mature forests will typically have a structure, composition and microclimate that is more similar to mature forests than those further away (Baker *et al*., 2014; Bauld *et al*., 2023; Hughes *et al*., 2023). This suggests that while edge effects on structural metrics such as canopy height and stem density may be observed in regenerating forests, the mechanisms behind them are at least partly distinct from those operating at cleared mature forest edges. As a result, the structure of edges within regenerating forest is likely to be different to that of cleared edges, with boundaries more characteristic of ecotones showing a lower magnitude but greater depth of edge effects (Harper *et al*., 2005).

Moreover, as discussed above, we expect that the trajectory of edge development will generally differ in edges in recovering secondary forests compared to mature forests. Edge effects at the boundary of young regenerating forests may be relatively small, but could develop as the forest continues to age and patch contrast at the forest edge increases. Further research into the development and impact of edge effects within regenerating forests is needed to better characterize how their trajectories differ from edge development at cleared and maintained edges that have dominated much of the literature to date.

## VI. Using remote sensing to characterize forest edge effects across spatial, temporal and ecological scales

Testing the predictions of our framework requires the ability to track responses of forest edges over large spatial extents and long time periods. Historically, the majority of studies of forest edge responses have used field data from plots located along edge‐to‐interior gradients (Harper *et al*., 2005, 2015). These studies are typically short‐term and focus on one site or forest ecosystem type. Long‐term experiments, such as the Biological Dynamics of Forest Fragments Project (BDFFP), Wog Wog and the SAFE project, have generated detailed data on change at forest edges and have been hugely valuable in advancing our understanding of temporal responses to forest edge creation (Margules, 1992; Ewers *et al*., 2011; Laurance *et al*., 2018). However, these experiments are challenging to replicate on a wider scale.

Increasingly, remote sensing technologies are allowing us to track forest change at spatial scales and temporal resolutions that are challenging to match in the field. Structural parameters, such as canopy height, gap fraction and vertical vegetation profiles, can be easily and robustly derived from airborne LiDAR data across 10–100 km^2^ of forests in a single acquisition (Ordway & Asner, 2020; Dalagnol *et al*., 2021; Reis *et al*., 2022), while multi‐spectral satellite timeseries can be used to constrain estimates of edge age (Silva *et al*., 2020) and map variation in canopy functional composition (Aguirre‐Gutiérrez *et al*., 2021; Kamoske *et al*., 2022).

Together, these emerging data streams offer a unique opportunity to comprehensively test the predictions of our framework of forest edge development. Here, we outline four suites of metrics that can be measured with remote sensing approaches, which could be used to assess variation at forest edges in time and across environmental gradients.

### 1. Measuring changes in 3D structure and complexity

**Fig. 3:**
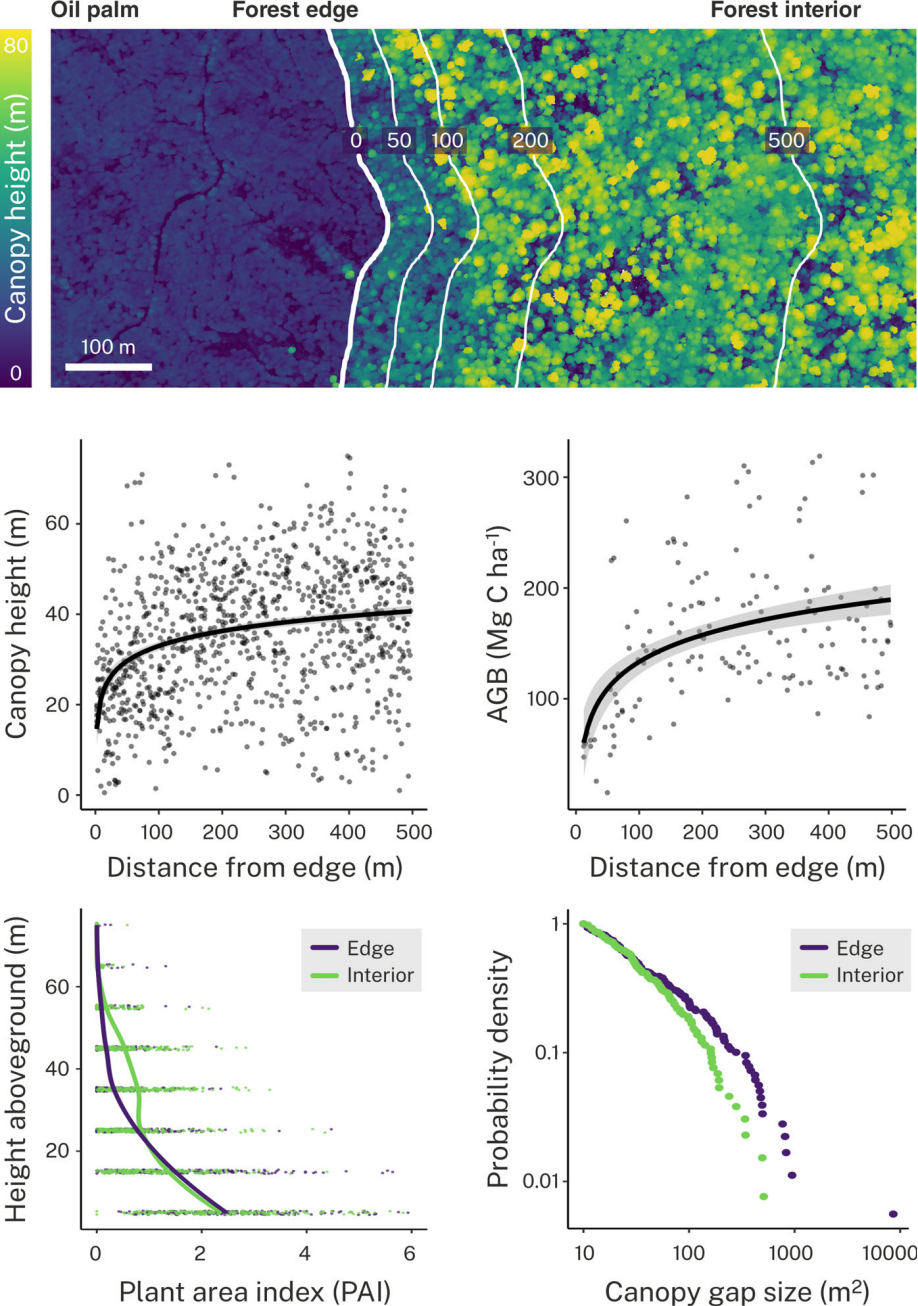
Real‐world example of how airborne LiDAR data can be used to advance our understanding of forest edge effects. The top panel shows a canopy height model of a transition zone from oil palm plantation to old‐growth forest in Malaysian Borneo. These data reveal strong declines in both canopy height and aboveground biomass (AGB) across this edge‐to‐interior gradient (middle panels), particularly in the 100–200 m closest to the edge. Additionally, we also observe major differences between forest edges and forest interiors (< 150 m and > 600 m from the edge, respectively) in terms of vertical and horizontal canopy 3D structure (bottom panels). In particular, the plant area index (PAI) of the emergent canopy and mid‐story was noticeably lower near forest edges. Moreover, forest edges had considerably more canopy gaps > 100 m^2^, which is approximately the size of the average tree crown in this system. For details of our methodology, see Supporting Information Notes S1.

As edges develop through time, we expect significant changes in stand organization (Fig. 2). Terrestrial, airborne and even spaceborne LiDAR data can provide high‐quality information on vertical structural heterogeneity (Dubayah *et al*., 2021; Kampe *et al*., 2010), allowing for large‐scale comparisons across sites and regions. In particular, LiDAR can be used to trace vertical vegetation density profiles using metrics such as plant area density (PAD), which estimates vegetation area per unit volume throughout the vertical profile of the canopy (Meeussen *et al*., 2020; Béland & Kobayashi, 2021). Metrics like PAD, therefore, provide an intuitive way to describe variation in canopy organization in space and time. Studies of forest edges have found distinct PAD profiles between forest interiors and edges, with forests near edges typically having higher PAD in the understorey but lower PAD in the taller parts of the canopy (Fig. 3; Almeida *et al*., 2019; Meeussen *et al*., 2020; Maeda *et al*., 2022), consistent with shifts in the vertical distribution of vegetation as forest edges develop. Monitoring changes in PAD profiles at forest edges relative to interior forest over time, or across a chronosequence using a space‐for‐time approach, could help to fingerprint the stages of forest edge development. Furthermore, because PAD profiles vary across different forest types, reflecting differences in canopy height, canopy openness and vertical complexity (Tang *et al*., 2016; Béland & Kobayashi, 2021), comparing PAD responses to edge creation across different forest types would help clarify how these are shaped by baseline differences in forest structure. These analyses, however, require careful methodological considerations to ensure PAD estimates are comparable across LiDAR acquisitions (Vincent *et al*., 2017; Zhang *et al*., 2024) and to control for variation in PAD across seasons (Nunes *et al*., 2022).

### 2. Measuring variation in large tree mortality and stand dynamics

A central prediction of our framework is that both mortality and growth rates should increase at forest edges, resulting in higher overall rates of stem turnover and a decline in the frequency of large trees. Repeat airborne LiDAR surveys provide an ideal vantage point from which to track forest canopy dynamics over time across broad spatial scales (Jackson *et al*., 2024). Canopy gap formation and dynamics can be precisely mapped using 3D canopy height models derived from LiDAR (Jucker, 2022). Rates of canopy gap formation have been shown to closely mirror variation in stand‐level tree mortality and turnover rates (Dalagnol *et al*., 2021; Lines *et al*., 2022), thus providing a tool to quantify changes in stem turnover and stand dynamism at forest edges. At the same time, these data can also be used to track canopy growth and structural recovery post‐disturbance (Chan *et al*., 2025), making it possible to monitor rates of forest edge recovery through time and in relation to distance from edge (Fig. 3). To complement this canopy‐scale approach, both LiDAR and high‐resolution imagery from drones and even satellites are being increasingly used in combination with crown segmentation algorithms to map individual trees and their demographic rates over time (Ball *et al*., 2023; Battison *et al*., 2024). While these individual‐based approaches do not translate well to understorey trees, especially in dense forests, they do provide a robust way to map the crowns of large, canopy‐dominant trees. As such, they could be used to track the dynamics of large canopy trees across edge‐to‐interior gradients over time, shedding new light on the extent and time frame over which these key individuals are impacted by edge formation.

### 3. Measuring shifts in canopy composition and function

Canopy structural changes occurring at forest edges are expected to occur in tandem with changes in tree species composition, such as increases in the proportion of pioneer species characterized by resource‐acquisitive traits and high turnover rates (Melito *et al*., 2018). These compositional changes are expected to be greater in moist, closed‐canopy forests with high structural complexity, where the proportion of shade‐tolerant species is higher than in more open forests (Ehbrecht *et al*., 2021). Capturing these shifts in species composition through time and along environmental gradients is inherently challenging on the ground. Airborne hyperspectral imagery provides an avenue to map variation in the functional traits and functional diversity of forest canopies at landscape and regional scales (Swinfield *et al*., 2020a; Aguirre‐Gutiérrez *et al*., 2021; Kamoske *et al*., 2022). This approach has been used to detect increases in foliar nutrient concentrations and declines in leaf mass area at forest edges in Borneo, consistent with increases in the proportion of fast‐growing pioneer species (Ordway *et al*., 2022). Increasing availability of high‐resolution, multispectral satellite archives enhances our ability to map canopy functional composition at global scales (Aguirre‐Gutiérrez *et al*., 2021). Combining these maps of canopy composition with structural information obtained using airborne or satellite LiDAR would enable the links between structural and compositional changes at forest edges to be studied explicitly across wider scales (Kamoske *et al*., 2021; Holcomb *et al*., 2024).

Moreover, spectral imagery that extends into the near and far infrared also provides a unique opportunity to capture the physiological responses driving changes at forest edges. Metrics such as canopy water content and evapotranspiration – which are directly related to plant hydraulic function and stress – and solar‐induced fluorescence – a proxy of photosynthetic capacity – can be derived from both airborne and satellite platforms and used to monitor variation in canopy health and productivity both through time and space (Asner *et al*., 2016; Konings *et al*., 2017, 2021; Yang *et al*., 2018; Fisher *et al*., 2020; Fancourt *et al*., 2022). Satellite‐derived measures of canopy water content have been used to show that canopy desiccation in dry season Amazonian forests is more pronounced at forest edges (Briant *et al*., 2010). Linking similar observations of changes in forest function with structural and compositional changes occurring at forest edges would offer important new insights into the underlying physiological mechanisms underpinning forest edge effects.

### 4. Measuring outcomes for forest aboveground biomass

A key area for further research is in clarifying changes in AGB at forest edges across a broader range of forest types (Smith *et al*., 2018). Field‐based forest inventories are the gold standard for estimating forest AGB, but are labour intensive and therefore typically only cover small spatial areas. Moreover, few permanent plots are intentionally established near forest edges, as this comes with a larger risk of losing long‐term data and infrastructure. Again, remote sensing‐derived estimates of forest AGB – particularly wall‐to‐wall maps generated using airborne LiDAR and point‐based estimates derived from satellites like GEDI (Meyer *et al*., 2013; Jucker *et al*., 2018a; Duncanson *et al*., 2022) – provide an obvious solution to this challenge. For example, Bauer *et al*. (2024) used GEDI data to quantify AGB declines at forest edges across the Amazon Basin, and showed that AGB losses at forest edges were higher in more fragmented landscapes. While caution is needed, in particular because the scale at which edge effects occur is often smaller than the resolution used to map AGB (Duncanson *et al*., 2025; Morreale *et al*., 2025), the application of remotely sensed AGB estimates across a broader range of forest landscapes would help in capturing large‐scale variation in AGB changes at forest edges across a variety of forest types. Further, these AGB estimates could be linked with environmental and climate data, as well as other remotely sensed structural metrics, such as canopy height and density, to test how factors related to patch contrast modulate edge effects in global forests.

## VII. Conclusions

Today, most of the world's forests grow in close proximity to an edge. But because edge effects have been observed to vary dramatically in their strength and even direction among studies, it remains unclear whether their impact on forest structure, composition and function is predictable or simply too highly context‐dependent. In this paper, we argued that trajectories of edge development are, in fact, broadly consistent across forest ecosystems. Divergent outcomes at forest edges between forest ecosystems can be largely explained by variation in their age, the climatic context and the structural baseline of the forest. Consequently, their temporal trajectories are broadly predictable once placed within the appropriate environmental context. Emerging remote sensing technologies offer a unique opportunity to put our framework to the test, providing a pathway to better integrate forest edge effects into our global understanding of the terrestrial carbon cycle.

## Competing interests

None declared.

## Author contributions

RBM and TJ conceived the idea for this review paper. RBM led the writing of the paper, with TJ contributing substantially to revisions.

## Disclaimer

The New Phytologist Foundation remains neutral with regard to jurisdictional claims in maps and in any institutional affiliations.

## Supporting information


**Notes S1** Methodology for creating Figure 3.
Please note: Wiley is not responsible for the content or functionality of any Supporting Information supplied by the authors. Any queries (other than missing material) should be directed to the *New Phytologist* Central Office.

## Data Availability

The data used to create Fig. 3, and information on the methodology used to generate estimates of canopy height, AGB and PAI, are publicly archived on Zenodo: https://zenodo.org/records/4020697 (Swinfield *et al*., 2020b).
