# Supplementary material for: A unifying framework for understanding how edge effects reshape the structure, composition and function of forests
Source: New Phytol. 2025 Aug 13;248(2):529–41. doi: 10.1111/nph.70457 (PMC12445831; doi:10.1111/nph.70457)
Supplement: Supplementary file 1 — Notes S1 Methodology for creating Figure 3. Please note: Wiley is not responsible for the content or functionality of any Supporting Information supplied by the authors. Any queries (other than missing material) should be directed to the New Phytologist Central Office. [file NPH-248-529-s001.pdf]

New Phytologist Supporting Information

Article Title: A unifying framework for understanding how edge effects reshape the structure, composition and function of forests

Authors: Rebecca Banbury Morgan and Tommaso Jucker

Article acceptance date: 16 July 2025

Notes S1: Methodology for creating Figure 3

Data on canopy height, aboveground biomass (AGB), and plant area index (PAI) for part of the SAFE project landscape in Malaysian Borneo were downloaded from the public archive on Zenodo: https://zenodo.org/records/4020697. Canopy height data are available at 1m resolution, PAI data at 20m resolution, and AGB data at 100m resolution.

We identified forest patches within the landscape using a classification algorithm, which identified forest patches as areas of forest over 25ha in size, with at least 10% of the area >10m in height in the canopy height model.

To create the middle panels of figure 3 (plots of canopy height and AGB with distance to edge), within the area identified as forest, we calculated the distance of each forest pixel to the nearest forest edge, at 5m and 100m resolution respectively.

To plot the effect of edge proximity on PAI, we classified pixels over 600m from the edge as interior forest, and pixels >150m from the edge as edge forest, and sampled 200 pixels across each group.

To estimate gap size frequency distributions across the landscape, we split the landscape into 1ha tiles, and selected all tiles 600-800m from the edge as interior forest tiles, and all tiles <150m from the edge as edge forest tiles. We randomly sampled 35ha of each forest type, and within these areas, we identified all canopy gaps, defined as areas >10m^2 where the canopy height was <10m.
